# Analgesic and Antidiarrheal Properties of Lappaconitine, Possibly Through Cyclooxygenase and µ‐Opioid Receptor Interaction Pathways: In Vivo and In Silico Studies

**DOI:** 10.1002/iid3.70373

**Published:** 2026-02-22

**Authors:** Shahid Shah, Arifa Akter, Salehin Sheikh, Razina Rouf, Raihan Chowdhury, Jannatul Ferdous, Md. Shimul Bhuia, Imam Hossain Rakib, Md. Zahid Hasan, Siddique Akber Ansari, Irfan Aamer Ansari, Muhammad Torequl Islam

**Affiliations:** ^1^ Department of Pharmacy Gopalganj Science and Technology University Gopalganj Bangladesh; ^2^ Bioinformatics and Drug Innovation Laboratory BioLuster Research Center Ltd. Gopalganj Dhaka Bangladesh; ^3^ Department of Biotechnology and Genetic Engineering Gopalganj Science and Technology University Gopalganj Bangladesh; ^4^ Department of Chemistry University of Rajshahi Rajshahi Bangladesh; ^5^ Department of Pharmaceutical Chemistry, College of Pharmacy King Saud University Riyadh Saudi Arabia; ^6^ Departmentof Drug Science and Technology Universityof Turin Turin Italy; ^7^ Pharmacy Discipline Khulna University Khulna Bangladesh

**Keywords:** ADMET properties, analgesic, antidiarrheal, diterpenoid alkaloid, lappaconitine

## Abstract

**Background:**

Lappaconitine (LAP) is a diterpenoid alkaloid with strong anti‐inflammatory activity. However, there is limited information on its gastroprotective effects. The present study aimed to explore the analgesic and antidiarrheal effects of LAP through in vivo and in silico studies.

**Methods:**

For this, LAP was administered orally to *Swiss* albino mice at doses of 1 and 4 mg/kg (bw). The analgesic effect was assessed using the acetic acid‐induced writhing test. Simultaneously, antidiarrheal activity was evaluated using the castor oil‐induced diarrheal secretion test in mice. In addition, an in silico molecular docking analysis was conducted to forecast the participation of cyclooxygenases (COXs) and the µ‐opioid receptor.

**Results:**

According to our in vivo findings, LAP and combination therapy (LAP + diclofenac sodium) significantly (*p* < 0.05) alleviated the number of writhing episodes in the experimental animals compared to the control group. Furthermore, in the castor oil‐induced diarrhea model, LAP and combination therapy (LAP + loperamide) significantly (*p* < 0.05) prolonged the onset time of diarrhea and reduced the number of diarrheal secretions. Besides, the molecular docking study suggested that LAP showed better binding affinity (−8.2 and −7.8 kcal/mol) with COX‐1 and COX‐2 enzymes, respectively. Likewise, LAP exhibited the highest binding score (–9.8 kcal/mol) with the µ‐opioid receptor. Moreover, LAP demonstrated high gastrointestinal absorption with low toxicity.

**Conclusion:**

Therefore, LAP exerts potential analgesic and antidiarrheal effects, as well as synergistic properties with diclofenac sodium and loperamide through the cyclooxygenase and MOR interaction pathways.

## Graphical abstract

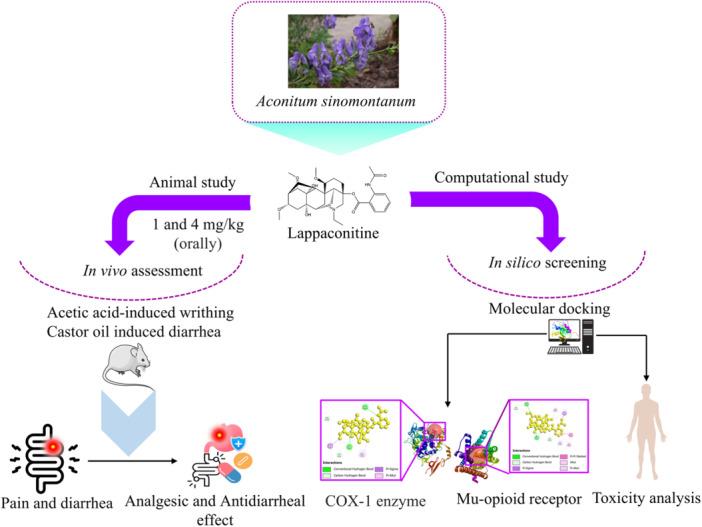

## 1 Introduction

The pain is an undesirable emotional and sensory experience that is related to tissue injury, either past or present [1]. It is frequently activated by painful stimuli and communicated to the central nervous system (CNS) via specialized neural networks, where it appears intrinsically [2]. According to the International Association for the Study of Pain (IASP), approximately one‐fifth (above 1 billion people) of the world's population suffers from chronic pain of varying degrees [3]. The physical, emotional, and overall quality of life of people are significantly impacted by chronic pain [4].

Numerous analgesics, including opioid analgesics, steroidal medications, and nonsteroidal anti‐inflammatory medicines (NSAIDs), have been employed to treat pain. NSAIDs reduce inflammation and discomfort by blocking the actions of cyclooxygenase (COX) enzymes, particularly COX‐2 [2]. But long‐term NSAID usage has been connected to a number of negative side effects, including the development of dormant obesity, skin atrophy, reduced density of bones, GIT ulcers, dependence, constipation, respiratory issues, inflammation of the liver, heart disease, kidney failure, erectile dysfunction, manic depression, high blood pressure, cramps, and fatigue [5, 6, 7].

On the other hand, diarrhea is a digestive ailment characterized by an atypical occurrence and consistency of bowel movement, which is prevalent in underdeveloped nations, particularly among children, and claims millions of victims globally [8, 9]. Diarrhea is mostly caused by pathogens, such as bacteria, parasites, or viruses, which are frequently consumed through unclean food or polluted water [10, 11]. Additionally, diarrhea is a familiar symptom of inflammatory bowel disease (IBD), such as ulcerative colitis and Crohn's disease [12]. Loperamide is commonly used to manage and alleviate the symptoms of acute diarrhea. It is also prescribed for treating chronic diarrhea in patients with IBD. The drug works by slowing intestinal movement, helping to reduce diarrhea. The use of loperamide is associated with several physiological problems, such as blood in the stools, constipation, fever, loss of appetite, nausea or vomiting, or stomach pain [13, 14]. Therefore, it is crucial that worldwide people have access to reasonably priced herbal alternatives for analgesics and antidiarrheal medications that are both more strong and less likely to cause side effects when taken from medicinal plants [15, 16]. Plants have a wide range of phytochemicals that have different biological effects on living things, which contributes to their therapeutic potential [17, 18].

Lappaconitine (LAP: C_32_H_44_N_2_O_8_) is a diterpenoid alkaloid obtained from the roots of *Aconitum sinomontanum* Nakai, which belong to the Aconite family [19]. Which has a wide range of pharmacological activities, including anti‐hypersensitivity [20], antibacterial [21], anticancer [22], antiarrhythmic [23], antinociceptive [24], analgesic, and anti‐inflammatory [25, 26]. According to Li et al. the low‐toxic LAP derivatives have potential analgesic activities [4]. Also, Qu et al. reported that in Chinese cancer patients found LAP to be an excellent non‐addictive medication for the management of moderate pain and postoperative pain. Its analgesic impact (ED_50_ = 3.5 mg/kg) is seven times more than that of aminopyrine and comparable to morphine and pethidine [27].

Molecular docking is a significant tool for generating designs and layouts of novel medications. It is predicted that a tiny molecule will exhibit affinity and attach experimentally to the binding location of the target receptor [28]. A good docking system must accurately predict the native ligand modeling to the receptor binding region and the related physicochemical molecular reactions [29]. Because medications have considerable side effects and possible toxicity, it is necessary to analyze the adsorption, distribution, metabolism, excretion, and toxicity (ADMET) properties during the drug manufacturing process. These early screening initiatives can boost success rates and shorten the time required to evaluate applicants for pharmaceuticals [30]. The primary objective of this study was to investigate the analgesic and antidiarrheal activities of LAP using two behavioral assays: the acetic acid‐induced writhing test and the castor oil‐induced diarrheal secretion model, conducted on *Swiss* albino mice. Additionally, an in silico analysis was performed to identify potential targets underlying the analgesic and antidiarrheal mechanisms and to predict the pharmacokinetic and toxicological properties of LAP.

## 2 Materials and Methods

### 2.1 In Vivo Study

#### 2.1.1 Chemicals and Reagents

The chemical LAP (CAS No: 32854‐75‐4, purity: 98% by HPLC) was obtained from Chengdu Alfa Biotechnology Co. Ltd. (China). The compounds diclofenac sodium (DFS) and loperamide (LOP) were purchased from Opsonin Pharma and Square Pharmaceuticals Ltd., Bangladesh, respectively. Tween 80, acquired from Merck (India), was used for dose preparation.

#### 2.1.2 Experimental Animals

The Animal House of Jahangirnagar University in Dhaka, Bangladesh, provided adult male *Mus musculus* (*Swiss* mice; average body weight: 24–30 g). These mice were randomly divided into five groups. Before the study, the animals were kept in standard conditions for 7 days (temperature: 26 ± 2°C, relative humidity: 65%). They had unrestricted access to standard food and water *ad libitum*. The Animal Ethics Committee of the Pharmacy Department at GSTU ([#GSTU/RC2024 (18PHR057)1]) approved this investigation.

#### 2.1.3 Study Design

##### 2.1.3.1 Acetic Acid‐Induced Writhing in Mice

For the assessment of analgesic activity, we performed an experiment according to Koster et al. In the procedure of the experiment, a total of 25 mice were divided into five groups, and each group contained five mice (*n* = 5). We treated mice (vehicle, LAP, and DFS) according to Table 1. Thirty minutes posttreatment, the mice were subjected to writhing caused by an intraperitoneal injection of 0.7% acetic acid at a dosage of 10 mL/kg of body weight. Each mouse was then placed inside a large glass cylinder, and the total number of writhing episodes occurring within a 10‐min period after the acetic acid injection was counted (visual counting) [31]. The percentage inhibition of writhing was determined using the following formula:

$$\%\;\text{Inhibition}=(1-\frac{\text{No}.\;\text{of Writhing}\;\left(\text{Test compound or Standard}\right)}{\text{No}.\text{of Writhing}\;\left(\text{Control}\right)})\times 100$$

**Table 1:** Analgesic and antidiarrheal experiment treatment groups with their details at 10 mL/kg volume of administration via oral route.

Treatment groups		Description	Administration design
Individual groups	Control	Vehicle: Distilled water containing 0.9% NaCl and 0.5% tween 80	Monotherapy (p.o)
	DFS‐25	Diclofenac sodium (COX inhibitor) at 25 mg/kg	Monotherapy (p.o)
	LOP‐3	Loperamide (µ‐opioid receptor agonist) at 3 mg/kg	Monotherapy (p.o)
	LAP‐1	Lappaconitine (test sample) at 1 mg/kg	Monotherapy (p.o)
	LAP‐4	Lappaconitine (test sample) at 4 mg/kg	Monotherapy (p.o)
Combination groups	DFS‐25 + LAP‐4	Diclofenac sodium 25 mg/kg + Lappaconitine 4 mg/kg	Sequential therapy (p.o)
	LOP‐3 + LAP‐4	Loperamide 3 mg/kg + Lappaconitine 4 mg/kg	Sequential therapy (p.o)

*Note:* Control: Vehicle (distilled water containing 0.9% NaCl and 0.5% tween 80). (*n* = 5).

Abbreviations: DFS, diclofenac sodium; LAP, lappaconitine; LOP, loperamide; p.o, Oral.

##### 2.1.3.2 Castor Oil‐Induced Diarrheal Secretion in Mice

For the assessment of antidiarrheal activity, we conducted an experiment according to Sharma et al. with a slight modification. In the procedure of the experiment, a total of 25 mice were divided into five groups, and each group contained five mice (*n* = 5). We treated mice (vehicle, LAP, and LOP) according to Table 1. Following 30 min of therapy, the mice were subjected to diarrheal secretion through the oral injection of castor oil (0.5 mL per mouse). The animals were then placed in adsorbent paper‐lined enclosures and observed for 4 h for the secretions of diarrhea (visual counting), which was defined as loose, watery stools. For every group, the total number of diarrheal secretions during 4 h and the first diarrheal stool (beginning of action) were noted [32]. The percentage inhibition of diarrheal secretions was determined using the following formula:

$$\%\;\text{Inhibition}=(1-\frac{\text{No}.\;\text{of diarrheal secretion}\;\left(\text{Test compound or Standard}\right)}{\text{No}.\;\text{of diarrheal secretion}\;(\text{Control})})\times 100$$

### 2.2 Statistical Analysis

The analgesic and antidiarrheal activities results were given as the mean value accompanied by the standard error of the mean (SEM) (*n* = 5). One‐way ANOVA, succeeded by Student's *t*‐test with Newman–Keuls post hoc (two‐tailed) analysis for multiple comparisons at 95% confidence intervals, using Graph Pad Prism (version 9.0), a statistical software application. Significance was attributed to *p‐*values below 0.05.

### 2.3 In Silico Study

#### 2.3.1 Ligands Collection and Preparation

**Figure 1:**
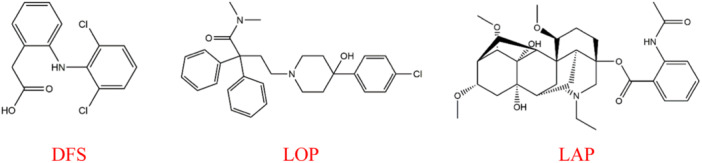
Chemical structures of test sample and standard drugs. DFS, diclofenac sodium; LAP, lappaconitine; LOP, loperamide.

The 3D chemical structures of the standard medications DFS (PubChem CID: 3033), LOP (PubChem CID: 3955), and the test compound LAP (PubChem CID: 441743) were obtained from the PubChem database. We reduced the internal energy of each ligand using Allinger's force field (MM2) approach with Chem3D Pro20.1.1 software [33]. The 2D chemical structures of LAP and the standard drugs DFS and LOP are shown in Figure 1.

#### 2.3.2 Macromolecules Collection and Preparation

**Figure 2:**
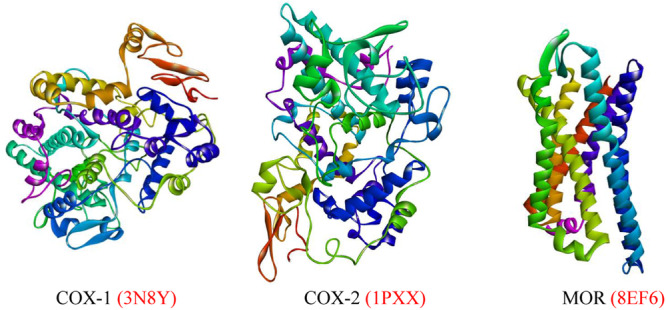
The 3D protein structure of COX‐1, COX‐2 enzyme and MOR. COX‐1, cyclooxygenase‐1; COX‐2, cyclooxygenase‐2; MOR, µ‐opioid receptor.

According to the literature review, we focused on three proteins that cause pain and diarrhea. The Research Collaboratory for Structural Bioinformatics Protein Data Bank was used to gather the 3D PDB structures of the targeted proteins COX‐1 (PDB ID: 3N8Y; Resolution: 2.60 Å) [34], COX‐2 (PDB ID: 1PXX; Resolution: 2.90 Å) [35], and µ‐opioid receptor (MOR) (PDB ID: 8EF6; Resolution: 3.20 Å) [36] (Figure 2). To ensure optimal function of the macromolecules, all unnecessary components, including lipids, water molecules, cocrystal molecules, and heteroatoms, were eliminated from the protein sequence using the PyMol software package (v2.4.1). The SwissPDB Viewer program was used to perform energy reduction and optimize the geometry of the macromolecules by employing the GROMOS96 force field and saving the PDB file to complete molecular docking [37].

#### 2.3.3 Molecular Docking and Visualization

Molecular docking was performed using PyRx virtual screening software (version 0.8). To expedite the docking procedure, the drugs were selected as ligands and the proteins as macromolecules with a grid box of maximum size. The docking potential output was stored in “.csv” format, and the ligand‐protein complex was saved in PDB format for ligand collection in PDBQT format [38]. The Discovery Studio Visualizer (v24.1.0.23298) and PyMol (v2.4.1) program packages were used to observe the nonbond interactions between the ligand‐receptors and the receptor's active site [39].

#### 2.3.4 Pharmacokinetic and Toxicity Prediction

Drug‐likeness is a qualitative assessment used in drug research and development to examine how chemical compounds perform with regard to specific drug‐like characteristics, such as bioavailability. It is closely related to ADMET [40]. We applied both SwissADME and ADMETlab 3.0 online tools to estimate the drug‐likeness and pharmacokinetic characteristics of LAP [41, 42]. Moreover, the toxicity profile of LAP was assessed using the ProTox 3.0 web server. The primary functions of this server were to examine properties such as hepatotoxicity, neurotoxicity, nephrotoxicity, respiratory toxicity, cardiotoxicity, carcinogenicity, immunotoxicity, mutagenicity, cytotoxicity, and nutritional toxicity [43].

## 3 Results

### 3.1 In Vivo Study

#### 3.1.1 Acetic Acid‐Induced Writhing Test in Mice

**Figure 3:**
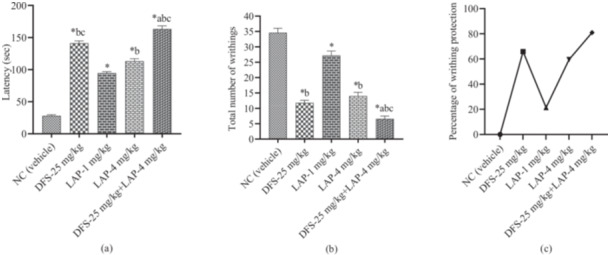
(a) Observed onset of writhing (sec), (b) Number of writhing, and (c) Percentage of writhing protection in different treatment and control groups (Values are [Mean ± SEM] [*n* = 5], One‐way ANOVA and *t‐*Student‐Neuman–Keuls post hoc test with multiple comparisons or *t‐*Student test, considering 95% confidence interval at *p* < 0.05; *p < 0.05, ^a^ *p* < 0.05, ^b^ *p* < 0.05, and ^c^ *p* < 0.05 when compared to the Vehicle (0.5% Tween‐80 in distilled water), DFS‐25, LAP‐1 mg/kg, and LAP‐4 mg/kg respectively; DFS, diclofenac sodium; LAP, lappaconitine; NC, negative control).

The result showed that LAP was dose‐dependent and significantly (*p* < 0.05) increased latency and reduced the number of writhing. The vehicle group exhibited short latency (28.00 ± 1.70 s) and the highest number of writhings (34.60 ± 1.43). Whereas the referral drug DFS (25 mg/kg) revealed better latency (141.20 ± 3.60 s) and a lower number of writhings (11.80 ± 0.86). Simultaneously, test sample LAP (1 and 4 mg/kg) promoted the latency and alleviated the number of writhings compared to the vehicle group. The LAP (4 mg/kg) showed good latency (113.20 ± 3.82 s) and reduced the writhing number (14.00 ± 1.22) compared to the LAP (1 mg/kg). Besides, the combination of DFS (25 mg/kg) and LAP (4 mg/kg) revealed the highest latency (163.40 ± 4.85 s) and the lowest number of writhing (6.60 ± 0.93) compared to the DFS (25 mg/kg) and LAP (4 mg/kg) treatment alone (Figure 3).

The DFS (25 mg/kg) showed relatively better writhing protection (65.90%). After that, the LAP (4 mg/kg) exhibited good writhing protection (59.54%). However, the combination group elevated the writhing protection (80.92%) compared to the DFS (25 mg/kg) and LAP (4 mg/kg) treatment alone. So, LAP (4 mg/kg) synergized the activity of DFS (25 mg/kg).

#### 3.1.2 Castor Oil‐Induced Diarrheal Secretion in Mice

**Figure 4:**
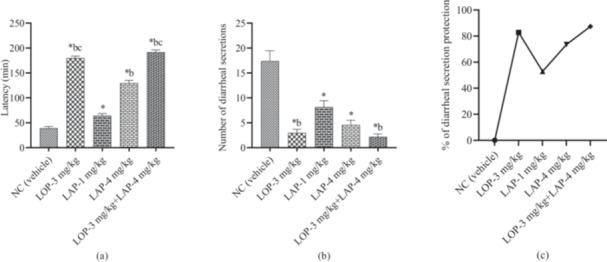
(a) Observed onset of diarrheal secretion (min), (b) Number of diarrheal secretion, and (c) Percentage of diarrheal secretion protection in different treatment and control groups (Values are [Mean ± SEM] [*n* = 5], One‐way ANOVA and *t‐*Student‐Neuman–Keuls post hoc test with multiple comparisons or *t‐*Student test, considering 95% confidence interval at *p* < 0.05; *p < 0.05, ^a^ *p* < 0.05, ^b^ *p* < 0.05, and ^c^ *p* < 0.05 when compared to the Vehicle [0.5% Tween‐80 in distilled water], LOP‐3, LAP‐1 mg/kg, and LAP‐4 mg/kg respectively; LAP, lappaconitine; LOP, loperamide; NC, negative control).

The result showed that LAP was dose‐dependent and significantly (*p* < 0.05) increased latency and reduced the hypersecretory diarrheal response. The vehicle group exhibited short latency (39.20 ± 3.65 min) and the highest number of diarrheal secretions (17.40 ± 2.06). Whereas the referral drug LOP (3 mg/kg) revealed better latency (180.00 ± 3.85 min) and a lower number of diarrheal secretions (3.00 ± 0.71). Simultaneously, test sample LAP (1 and 4 mg/kg) elevated the latency and alleviated the number of diarrheal secretions compared to the vehicle group. The LAP (4 mg/kg) showed good latency (129.60 ± 5.80 min) and reduced the number of diarrheal secretions (4.60 ± 0.93) compared to the LAP (1 mg/kg). Besides, the combination of LOP (3 mg/kg) and LAP (4 mg/kg) revealed the highest latency (192.00 ± 4.39 min) and the lowest number of diarrheal secretions (2.20 ± 0.58) compared to the LOP (3 mg/kg) and LAP (4 mg/kg) treatment alone (Figure 4).

The LOP (3 mg/kg) showed relatively better diarrheal secretions protection (82.76%). After that, the LAP (4 mg/kg) exhibited good diarrheal secretions protection (73.56%). However, the combination group elevated the diarrheal secretion protection (87.36%) compared to the LOP (3 mg/kg) and LAP (4 mg/kg) treatment alone. So, LAP (4 mg/kg) synergized with the activity of LOP (3 mg/kg).

### 3.2 In Silico Study

#### 3.2.1 Molecular Docking and Visualization

##### 3.2.1.1 DFS and LAP With Cyclooxygenase Enzyme Interaction

**Figure 5:**
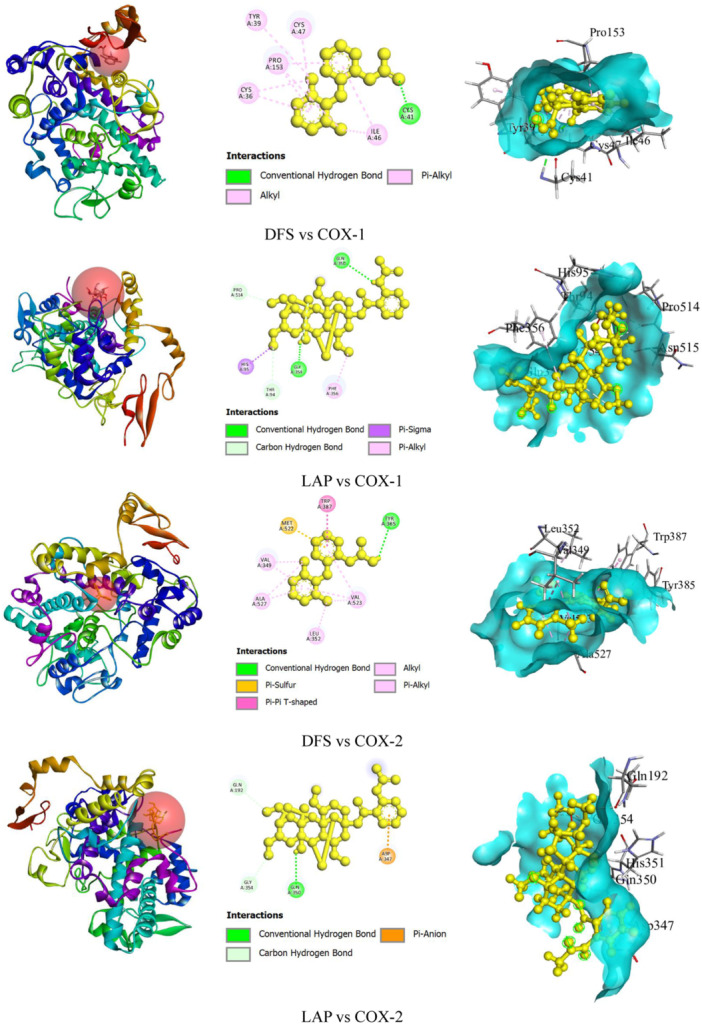
The ligand macromolecules complex and nonbond interaction amino acid residues LAP and DFS with COX‐1 and COX‐2 enzyme. COX‐1, cyclooxygenase‐1; COX‐2, cyclooxygenase‐2; DFS, diclofenac sodium; LAP, lappaconitine.

Our in silico investigation revealed that LAP showed the highest binding affinity (–8.2 kcal/mol) compared to DFS (–7.8 kcal/mol) against the COX‐1 enzyme (Table 2). Additionally, LAP formed five hydrogen bonds (HB) with GLN A:350, GLY A:354, PRO A:514, THR A:94, and GLY A:354 amino acid (AA) residues and hydrophobic (HP) bonds with HIS A:95 (Pi‐Sigma) and PHE A:356 (Pi‐Alkyl) AA residues. While DFS created one HB with CYS A: 41 AA residue and HP bonds with ILE A:46 (Alkyl), PRO A:153 (Alkyl), CYS A:36 (Alkyl), ILE A:46 (Pi‐Alkyl), PRO A:153 (Pi‐Alkyl), CYS A:36 (Pi‐Alkyl), CYS A:47 (Pi‐Alkyl), and TYR A:39 (Pi‐Alkyl) AA residues. Further, LAP exhibited lower binding energy (–7.8 kcal/mol) than DFS (–8.4 kcal/mol) against the COX‐2 enzyme. Moreover, LAP interacted with pain‐mediated enzyme COX‐2 to form three HB bonds with GLN A:350, GLN A:192, and GLY A:354 AA residues and an electrostatic bond with ASP A:347 (Pi‐Anion) AA residues. Whereas DFS formed one HB with TYR A:385 AA residue and HP bonds with MET A:522 (Pi‐Sulfur), TRP A:387 (Pi‐Pi T‐shaped), LEU A:352 (Alkyl), VAL A:523 (Alkyl), VAL A:349 (Alkyl), ALA A:527 (Alkyl), VAL A:523 (Pi‐Alkyl), VAL A:349 (Pi‐Alkyl), and ALA A:527 (Pi‐Alkyl) AA residues. Figure 5 shows the binding sites in both 2D and 3D perspectives.

**Table 2:** Docking scores and nonbond interaction amino acid residues of lappaconitine and diclofenac sodium with COX‐1 and COX‐2 enzymes.

Ligands	Enzymes	Docking scores (kcal/mol)	HB amino acid residues	Others bonding amino acid residues
DFS	COX‐1	–7.8	CYS A: 41	ILE A:46 (Alkyl) PRO A:153 (Alkyl) CYS A:36 (Alkyl) ILE A:46 (Pi‐Alkyl) PRO A:153 (Pi‐Alkyl) CYS A:36 (Pi‐Alkyl) CYS A:47 (Pi‐Alkyl) TYR A:39 (Pi‐Alkyl)
LAP		–8.2	GLN A:350 GLY A:354 PRO A:514 THR A:94 GLY A:354	HIS A:95 (Pi‐Sigma) PHE A:356 (Pi‐Alkyl)
DFS	COX‐2	–8.4	TYR A:385	MET A:522 (Pi‐Sulfur) TRP A:387 (Pi‐Pi T‐shaped) LEU A:352 (Alkyl) VAL A:523 (Alkyl) VAL A:349 (Alkyl) ALA A:527 (Alkyl) VAL A:523 (Pi‐Alkyl) VAL A:349 (Pi‐Alkyl) ALA A:527 (Pi‐Alkyl)
LAP		–7.8	GLN A:350 GLN A:192 GLY A:354	ASP A:347 (Pi‐Anion)

Abbreviations: COX‐1, cyclooxygenase‐1; COX‐2, cyclooxygenase‐2; DFS, diclofenac sodium; HB, hydrogen bond; LAP, lappaconitine.

##### 3.2.1.2 LOP and LAP With MOR Interaction

**Figure 6:**
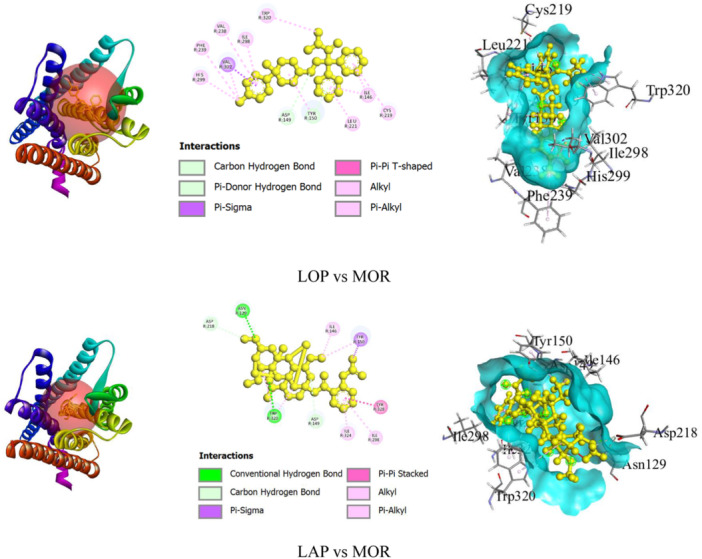
The ligand macromolecules complex and nonbond interaction amino acid residues LAP and LOP with MOR. LAP, lappaconitine; LOP, loperamide; MOR, µ‐opioid receptor.

Our in silico assessment exhibited that LAP showed excellent binding affinity (–9.8 kcal/mol) than LOP (–9.4 kcal/mol) connected with MOR (Table 3). Additionally, LAP formed four HBs with ASN R:129, TRP R:320, ASP R:218, and ASP R:149 AA residues and HP bond with TYR R:150 (Pi‐Sigma), TYR R:328 (Pi‐Pi Stacked), ILE R:146 (Alkyl), ILE R:298 (Pi‐Alkyl), ILE R:324 (Pi‐Alkyl), TYR R:150 (Pi‐Alkyl), and TRP R:320 (Pi‐Alkyl) AA residues. While LOP formed two HBs with ASP R:149 and TYR R:150 AA residues, as well as HP bonds with VAL R:302 (Pi‐Sigma), TYR R:150 (Pi‐Pi T‐shaped), VAL R:302 (Alkyl), ILE R:146 (Pi‐Alkyl), CYS R:219 (Pi‐Alkyl), LEU R:221 (Pi‐Alkyl), VAL R:238 (Pi‐Alkyl), ILE R:298 (Pi‐Alkyl), TYR R:150 (Pi‐Alkyl), PHE R:239 (Pi‐Alkyl), HIS R:299 (Pi‐Alkyl), and TRP R:320 (Pi‐Alkyl) AA residues. Figure 6 shows the binding sites in both 2D and 3D perspectives.

**Table 3:** Docking scores and nonbond interaction amino acid residues of lappaconitine and loperamide with µ‐opioid receptor.

Ligands	Enzymes	Docking scores (kcal/mol)	HB amino acid residues	Others bonding amino acid residues
LOP	MOR	–9.4	ASP R:149 TYR R:150	VAL R:302 (Pi‐Sigma) TYR R:150 (Pi‐Pi T‐shaped) VAL R:302 (Alkyl) ILE R:146 (Pi‐Alkyl) CYS R:219 (Pi‐Alkyl) LEU R:221 (Pi‐Alkyl) VAL R:238 (Pi‐Alkyl) ILE R:298 (Pi‐Alkyl) TYR R:150 (Pi‐Alkyl) PHE R:239 (Pi‐Alkyl) HIS R:299 (Pi‐Alkyl) TRP R:320 (Pi‐Alkyl)
LAP		–9.8	ASN R:129 TRP R:320 ASP R:218 ASP R:149	TYR R:150 (Pi‐Sigma) TYR R:328 (Pi‐Pi Stacked) ILE R:146 (Alkyl) ILE R:298 (Pi‐Alkyl) ILE R:324 (Pi‐Alkyl) TYR R:150 (Pi‐Alkyl) TRP R:320 (Pi‐Alkyl)

Abbreviations: HB, hydrogen bond; LAP, lappaconitine; LOP, loperamide; MOR, µ‐opioid receptor.

### 3.3 Pharmacokinetic and Toxicity Prediction

According to Table 4, LAP followed the Veber, Egan, and Muegge drug‐likeness rules. Further, gastrointestinal absorption (GIA) is high for all compounds, whereas DFS and LOP cross the blood‐brain barrier (BBB). They all have a bioavailability score of 0.55. LAP inhibits the CYP2D6 enzymes. The plasma protein binding (PPB) and half‐life of LAP were 68.2% and 1.73 h, respectively. LAP exhibited no adverse effects in hepatotoxicity, cardiotoxicity, carcinogenicity, mutagenicity, cytotoxicity, and nutritional toxicity endpoints. In contrast, LAP had toxic effects including neurotoxicity, nephrotoxicity, respiratory toxicity, and immunotoxicity.

**Table 4:** The documented summary of the in silico physiochemical, pharmacokinetic, and toxicityproperties of the potential diclofenac sodium, loperamide, and lappaconitine.

Properties	Parameters	Report/predicted value		
		DFS	LOP	LAP
Drug likeness	Lipinski	Yes; 0 violation	Yes; 1 violation: MLOGP > 4.15	Yes; 1 violation: MW > 500
	Ghose	Yes	No; 1 violation: MR > 130	No; 3 violations: MW > 480, MR > 130, #atoms> 70
	Veber	Yes	Yes	Yes
	Egan	Yes	Yes	Yes
	Muegge	Yes	No; 1 violation: XLOGP3 > 5	Yes
Pharmacokinetics	GI absorption	High	High	High
	BBB permeant	Yes	Yes	No
	Pgp substrate	No	Yes	Yes
	CYP1A2 inhibitor	Yes	Yes	No
	CYP2C19 inhibitor	Yes	Yes	No
	CYP2D6 inhibitor	Yes	Yes	Yes
	CYP3A4 inhibitor	No	Yes	No
	Bioavailability score	0.55	0.55	0.55
	PPB	99.2%	97.6%	68.2%
	CL_plasma_ (mL/min/kg)	0.46	6.29	3.76
	T_1/2_ (h)	1.64	0.151	1.73
Toxicity	Hepatotoxicity	Active	Inactive	Inactive
	Neurotoxicity	Active	Active	Active
	Nephrotoxicity	Active	Inactive	Active
	Respiratory toxicity	Active	Active	Active
	Cardiotoxicity	Active	Inactive	Inactive
	Carcinogenicity	Inactive	Inactive	Inactive
	Immunotoxicity	Inactive	Inactive	Active
	Mutagenicity	Inactive	Inactive	Inactive
	Cytotoxicity	Inactive	Inactive	Inactive
	Nutritional toxicity	Inactive	Inactive	Inactive

Abbreviations: BBB, blood‐brain barrier; CL, clearance; DFS, diclofenac sodium; GI, gastrointestinal; LAP, lappaconitine; LOP, loperamide; PPB, plasma protein binding; T_1/2_, half‐life.

## 4 Discussion

Pain is characterized as an unpleasant feeling that is connected to damaging external stimuli that have the potential to cause tissue damage [44]. Usually, endogenous inflammatory mediators such as histamine, bradykinin, prostaglandin, and serotonin cause pain. These chemicals increase peripheral nerve impulses, which causes nociceptors to become activated [45, 46]. The well‐researched acetic acid‐induced writhing test offers proof that peripheral systems are engaged. Treatment with acetic acid irritates the tissue, activating phospholipase A2 and releasing arachidonic acid from membrane phospholipids [47]. The enzyme COX subsequently transforms this arachidonic acid into prostaglandins. Prostaglandins, in particular PGE2, increase the experience of pain by sensitizing nociceptors, or pain receptors in peripheral tissues [48, 49, 50]. Additionally, acetic acid causes the activation and subsequent release of a number of inflammatory mediators and cytokines, including TNF‐α, IL‐8, and interleukin‐1β [51]. These chemical messengers are mostly produced by resident peritoneal macrophages and mast cells [52].

The present study's findings showed that the oral administration of LAP significantly (*p* < 0.05) decreased the number of abdominal writhing during the acetic acid‐induced writhing test. In particular, LAP (4 mg/kg) significantly (*p* < 0.05) decreased the writhing (59.54%), and combined with the DFS (25 mg/kg) group, dramatically alleviated the writhing (80.92%) more than the control and other groups in the experimental animals. So, LAP has analgesic characteristics. The way in which LAP has an analgesic effect is dose‐dependent manner [53]. The investigation by Zhang et al. also showed that oral LAP had a noticeably higher analgesic effectiveness than oral morphine, offering a new alternative for managing moderate to severe pain [54].

According to Gottlieb et al. [55], diarrhea is an irregular bowel movement that is characterized by an increase in the volume, frequency, and water content of stools. It is also often described clinically as a rise in the frequency of stools to three or more liquid or semi‐formed movements in a 24‐h period [55]. The present research conducted on castor oil induced in diarrheal mice, found a substantial reduction in the number, mass, and frequency of diarrheal stools, a reduction in intestinal fluid volume, gastrointestinal secretions of electrolytes, along with suppression of the peristaltic index. Ricinoleic acid particularly exists in castor oil, irritates the intestinal mucosa, promotes gastrointestinal release, and increases intestinal peristalsis [56]. Additionally, ricinoleic acid increases the production of nitric oxide (NO) and adenylate cyclase by epithelial cells, which raises the production of cyclic adenosine monophosphate (cAMP) [57]. According to reports, increased cAMP levels reduce the absorption of sodium and potassium ions, cause peristalsis, change the practicality of membranes, and decrease the activity of the Na^+^/K^+^‐ATPase pump in the intestinal wall. All of these effects lead to the creation of electrolytes and the accumulation of water in the gut [58, 59]. Because of its excessive production in abnormal situations [60]. NO is thought to be a key pro‐inflammatory cytokine that causes inflammation. NO also causes instantaneous secretory effects by opening chloride channels [61]. The primary receptors implicated in diarrheal processes are calcium channel blockers, MOR, COX‐1, COX‐2, and α1‐adrenoceptors. Colonic movement is delayed when the MOR is activated [8]; By constricting the smooth muscle cells of the gastrointestinal tract (GIT), calcium channel blockers lessen the frequency and intensity of frequent bowel movements [62]; By preventing COX‐2 from secreting prostaglandins, diarrheal discharges are decreased [63]. Thus, it is thought that ricinoleate‐induced diarrhea can be prevented by inhibiting prostaglandin production [64].

In the present in vivo experiment, LAP significantly (*p* < 0.05) decreased the diarrheal secretion and enhanced the latency period in comparison to the control group. At high doses, LAP showed the best antidiarrheal properties. Previous findings revealed LAP was isolated from A. leave Royle and demonstrated anti‐inflammatory and antinociceptive actions. The co‐administration of LAP and LOP provided superior overall protection against diarrheal discharges, exceeding the individual effects of each medication. Furthermore, since prostaglandins, along with acetylcholine, are recognized secretagogues—substances that encourage cAMP to release ions—LOP prevents their release [65]. This indicates a cooperative impact between LAP and LOP, which improves their collective effectiveness in treating diarrhea.

**Figure 7:**
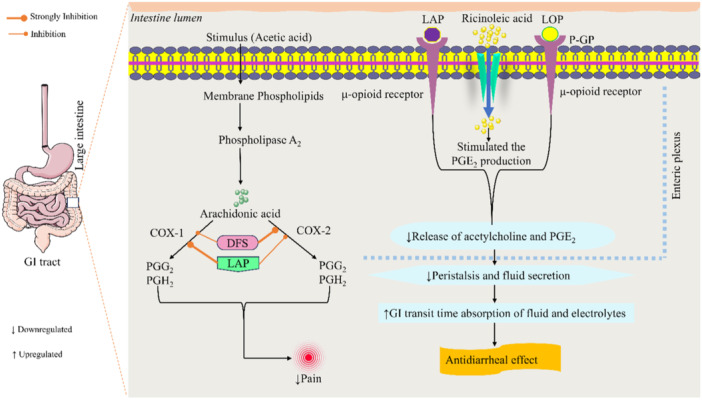
The analgesic and antidiarrheal mechanism of lappaconitine.

The process of discovering and creating new drugs is challenging, complex, and time‐consuming. In silico studies have become more widely accepted as a part of computer‐assisted drug development in recent years [66]. Molecular docking serves a vital role in the discovery of new medications and aids in understanding the drug's potential efficacy and selectivity by anticipating the molecular‐level interactions among tiny molecules and their target proteins [67]. According to our findings, LAP had a greater affinity for the COX‐1 enzyme (–8.2 kcal/mol) in comparison to DFS (–7.8 kcal/mol). LAP formed five HBs and HP bonds, whereas DFS formed a smaller number of HBs and a more diverse range of HP bonds. In the case of the COX‐2 enzyme, LAP exhibited better binding affinity –7.8 kcal/mol in comparison with DFS's (–8.4 kcal/mol). LAP also produced three HBs along with an electrostatic bond, whereas DFS only formed a single HB and had a significant HP bond. These findings indicate that LAP has a higher affinity for both COX‐1 and COX‐2 enzymes compared to DFS, also suggesting that LAP may have greater inhibitory effects. The variable binding patterns observed between LAP and DFS are indicative of their unique interaction processes with these enzymes [68]. However, LAP exhibited higher binding affinity (–9.8 kcal/mol) compared to standard drug LOP (–9.4 kcal/mol) connected with MOR. LAP interacted with MOR through HBs and HP bonds. On the other hand, the binding of LOP is characterized by a complex network of hydrogen bonding interactions. Overall, LAP has a higher binding affinity and interaction profile, which might potentially boost its therapeutic efficacy. The finding‐based analgesic and antidiarrheal mechanisms of LAP are illustrated in Figure 7.

During the first phases of drug development, drug‐likeness is used to offer informative suggestions, increasing the likelihood that a chemical will meet the requirements and be authorized for clinical trials [69]. A drug candidate has to fulfill Lipinski's five requirements, which include having a MW of 500 g/mol or less, a lipophilicity (LogP_o/w_) of five or less, a maximum of than five HBD, and no exceeding ten HBA [70]. We assess the drug‐like properties and ADMET characteristics of LAP using web‐based tools SwissADME and ProTox‐3.0. As seen in Table 4, every expected parameter for LAP (drug‐like characteristics and ADMET profile) stayed within permissible limits. This implies that LAP is rather safe when taken in large quantities. LAP exhibited no adverse effects in hepatotoxicity, cardiotoxicity, carcinogenicity, mutagenicity, cytotoxicity, and nutritional toxicity endpoints. In contrast, LAP may induce neurotoxicity, nephrotoxicity, respiratory toxicity, and immunotoxicity.

There are several restrictions on the research. First of all, the findings may not be as broadly applicable as they may be because of the limited sample size. Second, the research was done on animals, thus, the results may not be entirely applicable to humans. Third, a critical component of comprehending the safety profile of LAP is knowing its long‐term consequences, which were not investigated in this research. Fourthly, the in silico studies mainly focused on COX‐1, COX‐2, and MOR, which may not be sufficient to claim an extract molecular mechanism behind the LAP effect on pain and diarrhea. Furthermore, toxicity and other adverse effects were not thoroughly examined. Future studies should undertake long‐term investigations, bigger, more varied sample sizes, and clinical trials in order to properly determine the safety and effectiveness of LAP in humans and address these limitations.

Taken together, in vivo assessment, LAP showed analgesic activity, while combination therapy of (LAP + DFS) exhibited potential synergistic analgesic effects. In another in vivo study, LAP showed antidiarrheal activity, while combination therapy of (LAP + LOP) exhibited potential synergistic antidiarrheal effect. The in silico study revealed that LAP has a good binding score (–8.2 and –7.8 kcal/mol) with the COX‐1 and COX‐2 enzymes, respectively, as well as an excellent binding score (–9.8 kcal/mol) with the MOR. Additionally, it has acceptable toxicity criteria. Therefore, LAP exerts an analgesic and antidiarrheal effect and also synergistic properties with DFS and LOP through the COX and MOR interaction pathways.

## 5 Conclusions

Our study revealed that LAP and combination therapy (LAP + DFS) significantly (*p* < 0.05) reduced the number of writhing episodes in experimental animals compared to the control group. Additionally, LAP and the combination therapy (LAP + LOP) demonstrated significant antidiarrheal activity; both groups increased diarrheal latency and reduced diarrheal secretions in castor oil‐induced diarrhea models. Furthermore, LAP exhibited relatively good binding affinity with COX‐1 (−8.2 kcal/mol), COX‐2 (−7.8 kcal/mol), and MOR (−9.8 kcal/mol). ADMET analysis showed that LAP has high GIA and inhibits the CYP2D6 enzyme. LAP exhibits no harmful effects in many crucial toxicity measures, except for neurotoxicity, nephrotoxicity, pulmonary toxicity, and immunotoxicity. Therefore, LAP demonstrates potential analgesic and antidiarrheal effects, as well as synergistic properties with DFS and LOP through interactions with the COXs and MOR pathways. Future research should include detailed toxicological studies to refine its safety profile and establish safe dosing guidelines. Clinical trials are necessary to confirm these findings and validate LAP's efficacy and safety in human subjects.

## Author Contributions

**Shahid Shah:** resources, software, supervision, visualization, writing – original draft, writing – review and editing. **Arifa Akter:** resources, software, supervision, writing – original draft, writing – review and editing. **Salehin Sheikh:** investigation, methodology, writing – original draft, writing – review and editing. **Razina Rouf:** conceptualization, formal analysis, methodology, project administration, supervision, validation, writing – original draft, writing – review and editing. **Raihan Chowdhury:** conceptualization, data curation, formal analysis, investigation, methodology, project administration, resources, software, supervision, validation, visualization, writing – original draft, writing – review and editing. **Jannatul Ferdous:** validation, writing – original draft. **Md Shimul Bhuia:** writing – original draft, writing – review and editing. **Imam Hossain Rakib:** investigation, resources, software. **Md Zahid Hasan:** software, visualization, writing – original draft, writing – review and editing. **Siddique Akber Ansari:** writing – original draft, writing – review and editing. **Irfan Aamer Ansari:** writing – original draft, writing – review and editing. **Muhammad Torequl Islam:** conceptualization, data curation, formal analysis, investigation, methodology, project administration, resources, software, supervision, validation, visualization, writing – original draft, writing – review and editing.

## Funding

The authors received no specific funding for this work.

## Ethics Statement

This study was approved by the Animal Ethics Committee of Gopalganj Science and Technology University, Gopalganj Department of Pharmacy ([#GSTUSTU/RC2024 (18PHR057)1]). No anesthetic or surgical procedures were used for this study. We checked only the behavioral parameters after the treatment; therefore, this study did not require any medication to overcome or reduce the suffering of the experimental animals.

## Conflicts of Interest

The authors declare no conflicts of interest.

## Data Availability

The data that support the findings of this study are available from the corresponding author upon reasonable request.
